# The global proportion and volume of unrecorded alcohol in 2015

**DOI:** 10.7189/jogh.09.010421

**Published:** 2019-06

**Authors:** Charlotte Probst, Alexandra Fleischmann, Gerhard Gmel, Vladimir Poznyak, Dag Rekve, Leanne Riley, Margaret Rylett, Kevin D Shield, Jürgen Rehm

**Affiliations:** 1 Institute for Mental Health Policy Research, Centre for Addiction and Mental Health (CAMH), Toronto, Ontario, Canada; 2 Institute for Clinical Psychology and Psychotherapy, Technische Universität Dresden, Dresden, Germany; 3 World Health Organization/Pan-American Health Organization Collaborating Centre in Addiction and Mental Health, Toronto, Ontario, Canada; 4 Management of Substance Abuse, World Health Organization, Geneva, Switzerland; 5 Addiction Switzerland, Lausanne, Switzerland; 6 Alcohol Treatment Centre, Lausanne University Hospital, Lausanne, Switzerland; 7 University of the West of England, Bristol, United Kingdom; 8 Prevention of Noncommunicable Diseases, World Health Organization, Geneva, Switzerland; 9 Campbell Family Mental Health Research Institute, CAMH, Toronto, Ontario, Canada; 10 Institute of Medical Science, University of Toronto, Medical Sciences Building, Toronto, Ontario, Canada; 11 Department of Psychiatry, University of Toronto, Toronto, Ontario, Canada; 12 Dalla Lana School of Public Health, University of Toronto, Toronto, Ontario, Canada

## Abstract

**Background:**

Alcohol consumption is associated with elevated risks of disease and injury, and the best indicator of the level of consumption in a country is total alcohol *per capita* (APC) consumption among adults which comprises recorded consumption and unrecorded consumption. While recorded consumption can be assessed with small measurement bias via taxation or other governmental records, unrecorded consumption is more difficult to assess. The objectives of this study were to estimate the country-specific proportion and volume of unrecorded APC in 2015, to identify main sources of unrecorded alcohol and to assess to what extent experts perceive unrecorded alcohol as a public health, social, and financial problem.

**Methods:**

Estimates of unrecorded APC were based on a multilevel fractional response regression model using data from World Health Organization’s (WHO) STEPwise approach to surveillance surveys (16 countries, 66 188 participants), estimates from the routine WHO reporting on key indicators of alcohol use (189 countries), and a nominal group expert assessment (42 countries, 129 experts). Expert assessments also included data on the sources of unrecorded alcohol and the perception of unrecorded alcohol as a public health, social, and financial problem.

**Results:**

The volume of global unrecorded APC was 1.6 L pure alcohol, representing 25% of the total APC. The volume of unrecorded APC was highest in Europe (2.1 L *per capita*), while the proportion of unrecorded APC was highest in the WHO Eastern Mediterranean region (57% of the total alcohol). In countries with available data, homemade alcohol was identified as a major source of unrecorded alcohol. The majority of experts considered unrecorded alcohol to be a public health (62%), social (60%), and financial problem (54%).

**Conclusions:**

High volumes of unrecorded alcohol are consumed globally; however, the volumes consumed and the sources of the unrecorded alcohol exhibit large geographical variation.

Alcohol has been identified as a leading contributor to the burden of disease in all global comparative risk assessments to date [1-3]. As indicated by the World Health Organization’s (WHO) Global strategy to reduce the harmful use of alcohol [4], and the United Nations’ Sustainable Development Goals [5,6], the most important indicator of alcohol consumption at the national level is adult *per capita* consumption [7-9]. Total adult alcohol *per capita* (APC) consumption is comprised of two main categories: recorded consumption, which includes alcohol that is covered in routine statistics (mainly via taxation), and unrecorded consumption [10]. Unrecorded alcohol comes from a broad range of sources which vary by country [11], and it is typically defined as all alcohol which is consumed but not registered in a given country [12]. Namely, the sources include home production, surrogate alcohol (ie, alcohol not intended for human consumption such as mouthwash, cologne, or antifreeze), counterfeit (ie, homemade or illegally produced alcohol that is sold in replica bottles suggesting a certain brand) and other illegally produced alcohol (eg, alcohol that is produced in factories without declaring the production to the authorities), and alcohol that is produced in another jurisdiction and brought across the border (eg, cross-border shopping or smuggling). Furthermore, unrecorded alcohol is commonly the cheapest form of alcohol [11,13,14], and thus has been associated with heavy drinking patterns [15,16]. With the exception of cross-border shopping, unrecorded alcohol is often consumed by more vulnerable populations, such as people of low socioeconomic status, rural populations, and people with alcohol dependence [12]. Unrecorded alcohol may contain additional toxic compounds, such as methanol, disinfectants or heavy metals [11]. Both heavy drinking patterns and toxic compounds contribute to subsequent harms, particularly among vulnerable populations [16-18].

Globally, unrecorded alcohol has been estimated to represent approximately one-fourth of the total APC consumed [2]; however, the relative proportion of unrecorded APC to the total APC consumption is highest in low-income and lower-middle-income countries [19], where in several instances unrecorded alcohol makes up the majority of all alcohol consumed [2,19]. The measurement of unrecorded consumption has been limited since existing studies are out of date and use non-systematic methodologies, resulting in national volumes of unrecorded consumption being estimated via expert judgement [10,20], except in those few countries (such as the Nordic countries) which conduct regular surveys and/or studies to estimate unrecorded consumption [21]. National studies have used various methodologies, such as estimating total consumption indirectly from health markers, such as alcohol poisoning rates [22,23]; however, these methods cannot systemically monitor unrecorded consumption at the global level, as different markers are linked to total consumption in different countries. Thus, there is a critical need to better understand the level of unrecorded consumption in all countries in order to determine appropriate policy measures and provide an efficient allocation of resources. The overall aim of this study was to estimate the country-specific volumes of unrecorded alcohol consumption for 2015. Furthermore, specific aims of the study were to investigate the relative importance of different sources of unrecorded alcohol on the country level as well as the perception of unrecorded alcohol as a public health, financial, or social problem.

## METHODS

A prediction model was fitted in order to estimate the country-specific proportion of unrecorded alcohol of the total APC consumed among adults in 2015 [24]. A series of country level predictors for the year 2015 was available for the model building based on observed data on the country-specific proportion of unrecorded alcohol from three different sources. The litres of recorded APC in 2015 [25] were then used to calculate the volume of unrecorded APC consumed at the country level (in litres of pure alcohol). All study methods adhered to the Guidelines for Accurate and Transparent Health Estimates Reporting (GATHER statement; S5 Table) [26].

### Data sources

To improve the surveillance of alcohol consumption, and of unrecorded consumption in particular, a module to measure unrecorded consumption was incorporated into the WHO’s STEPwise approach to surveillance (STEPS) surveys starting in 2013 with a total of 66 188 participants (S1 Table) [27]. All surveys were designed to be nationally representative of adults 18 to 69 years of age and older. Quantity and frequency questions were available for total and unrecorded alcohol consumption in the past 7 days (Appendix S2 in **Online Supplementary Document**). The proportion of unrecorded alcohol consumed was calculated as the average APC consumption of unrecorded alcohol divided by the average total APC, taking survey weights into account.

A nominal group expert assessment was conducted between August 2015 and July 2016 [28,29], which assessed the proportion of unrecorded alcohol consumption as well as the perception of unrecorded alcohol as a public health, financial, or social problem in 49 WHO Member States. In total, 643 experts from 49 countries were contacted; 129 of these experts from 42 countries participated in the study. Data obtained from the nominal group expert assessment included information on the sources of unrecorded alcohol (data for the Democratic Republic of Congo were missing) as well as the perception of unrecorded alcohol as a public health, financial, or social problem (available for all 42 countries). Details of the methodology of this nominal group expert assessment can be found elsewhere [30] and in Appendix S2 in **Online Supplementary Document**. Details of the instrument used in the most recent nominal group expert assessment, which is very similar to the one previously used, are provided in Appendix S3 in **Online Supplementary Document**.

Additionally, estimates of the proportions of unrecorded alcohol in 189 countries were obtained from the WHO as part of their routine reporting of non-communicable disease indicators [31]. As most countries have not historically monitored unrecorded consumption, the WHO relied on expert judgements to obtain these estimates. Experts were asked if any changes in unrecorded consumption had occurred since the 2010 estimates of the last Global Status Report on Alcohol and Health [2], and, if so, the magnitude of the changes together with documented evidence supporting such changes. In addition, nominal group expert assessments had been conducted [28,29], assessing the proportion of unrecorded alcohol in 34 WHO Member States where unrecorded alcohol played a major role [19].

The following country level predictors were available for analysis. Data for 2015 on the proportion of urbanization, the prevalence of malnutrition, coverage of sanitation, and level of education were obtained from the Institute for Health Metrics and Evaluation for all except eight countries [32]. The *per capita* gross domestic product adjusted for purchasing power parity and respective income classifications for 2015 were obtained from the World Bank for all except five countries [33]. Information on country level alcohol statistics for 2015 (ie, the prevalence of drinking, recorded litres of APC per year, patterns of drinking scores, value added and excise taxation of alcoholic beverages, presence of a written national alcohol policy, presence of national legislation to prevent illegal production and/or sale of home or informally produced alcoholic beverages, and alcohol prohibition measures) were obtained from the Global Information System on Alcohol and Health [25], and from Al-Ansari et al. [34]. For 20 countries at least one of the alcohol policy indicators was missing.

### Statistical modelling

The country level proportions of unrecorded alcohol consumption were then predicted using a multilevel fractional response regression model, accounting for clustering of data points within countries [35,36]. Univariate models were fitted for all predictors. A first complete model was generated using a significance cut-off level of α<0.2 for predictor inclusion [37]. Predictors were further selected in a stepwise backward selection process combined with out-of-sample predictions (multiple random 10% sub-samples) and plausibility checks. The predictive precision was evaluated using R^2^ (ie, the proportion of the observed variance that is explained by the prediction model) [24].

The fitted regression model was then used to predict the proportion of unrecorded alcohol in all countries using covariate data. Confidence intervals of predicted values were calculated using the standard errors of predictions. The prediction of unrecorded alcohol consumption in 2015 was possible for 169 countries; unrecorded alcohol consumption was not estimated for 27 countries where information for one or more covariates was missing. The study results are presented for the five WHO regions and the four World Bank income groups (low-income, lower-middle-income, upper-middle-income, and high-income) [33]. In estimating a regional average, the proportion of unrecorded alcohol of the total APC was assumed to be the population-weighted average of all countries in the region.

Systematic differences across countries in the perception of unrecorded alcohol as a public health, financial, or social problem were investigated using logistic regression. All statistical analyses were performed using R version 3.3.1. The prediction model was fitted using the glmmPQL command (Fit Generalized Linear Mixed Models via PQL) of the nlme package [38]. The R source code used for all analyses can be obtained from the authors.

## RESULTS

The final prediction model explained about 70% of the variation in the observed data (R^2^ = 0.69). The model coefficients shown in **Table 1**. Socioeconomic indicators showed that a lower GDP PPP and higher levels of malnutrition were associated with a higher proportion of unrecorded alcohol. Lower levels of recorded alcohol were associated with a relatively higher proportion of unrecorded alcohol. The coefficients for alcohol policy indicators showed no significant association with the proportion of unrecorded alcohol after adjusting for all other factors in the model. A dummy variable regarding the data source showed that estimates based on survey data were associated with lower levels of unrecorded alcohol compared to nominal group expert assessments. When interpreting the coefficients it should be kept in mind that the model was fitted to optimize predictive precision and not to investigate the associations [39].

**Table 1:** Regression model used to estimate the proportion of unrecorded alcohol of the total alcohol *per capita* among adults in 2015

Predictor (reference)	β-coefficient	95% confidence interval		*P*-value
GDP PPP per capita*	-0.12	-0.20	-0.04	0.007
Malnutrition	2.56	0.65	4.47	0.006
No national alcohol policy	-0.08	-0.33	0.17	0.547
Alcohol is prohibited (no prohibition)	0.80	0.22	1.38	0.008
Litres of recorded alcohol	-0.14	-0.18	-0.09	<0.001
Recorded alcohol <1 L *per capita*	0.76	0.39	1.14	<0.001
**WHO region (European Region†):**				
African Region	-0.13	-0.51	0.25	0.493
Americas Region	-0.40	-0.75	-0.04	0.032
Eastern Mediterranean Region	-0.32	-0.90	0.27	0.301
South-East Asian Region	-0.31	-0.88	0.27	0.300
Western Pacific Region	-0.26	-0.67	0.15	0.220
Eastern Europe	0.87	0.26	1.48	0.006
**Data source (nominal group expert assessment):**				
Survey data	-1.00	-1.39	-0.61	<0.001
WHO estimates	-0.10	-0.28	0.08	0.295

GDP PPP – gross domestic product at purchasing power parity *per capita.* SE – standard error

*Refers to increments of 100 international dollars *per capita.*

†European Region, exclusive of eastern European countries (Belarus, Moldova, Russian Federation, Ukraine).

The volume of global unrecorded alcohol consumption in 2015 among adults was estimated to be 1.6 L of pure APC, representing 25% of all alcohol consumed (**Table 2**). The proportion of unrecorded APC of the total APC was highest in the Eastern Mediterranean (57% of total alcohol), while the volume of unrecorded consumption was highest in Europe (2.1 L *per capita*) (**Table 2**).

**Table 2:** Unrecorded consumption in 2015 by World Health Organization (WHO) regions and World Bank income groups

	Proportion unrecorded alcohol (%)	95% confidence interval		Volume unrecorded alcohol (litres)	Volume total alcohol (litres)
**WHO regions:**					
African Region	32.1	18.9	45.4	1.9	6.1
Region of the Americas	14.0	8.3	19.6	1.2	8.2
Eastern Mediterranean Region	56.8	41.0	72.6	0.3	0.6
European Region	19.8	11.5	28.1	2.1	10.6
South East Asian Region	44.7	28.6	60.9	2.0	4.5
Western Pacific Region	20.2	9.4	31.0	1.6	7.7
**Income groups:**					
Low-income	38.5	24.6	52.4	1.6	4.1
Lower-middle-income	41.7	26.5	56.8	1.9	4.6
Upper-middle-income	20.2	9.4	31.1	1.4	7.1
High-income	14.5	7.5	21.6	1.5	10.3
Global	24.5	9.9	39.2	1.6	6.7

**Figure 1:**
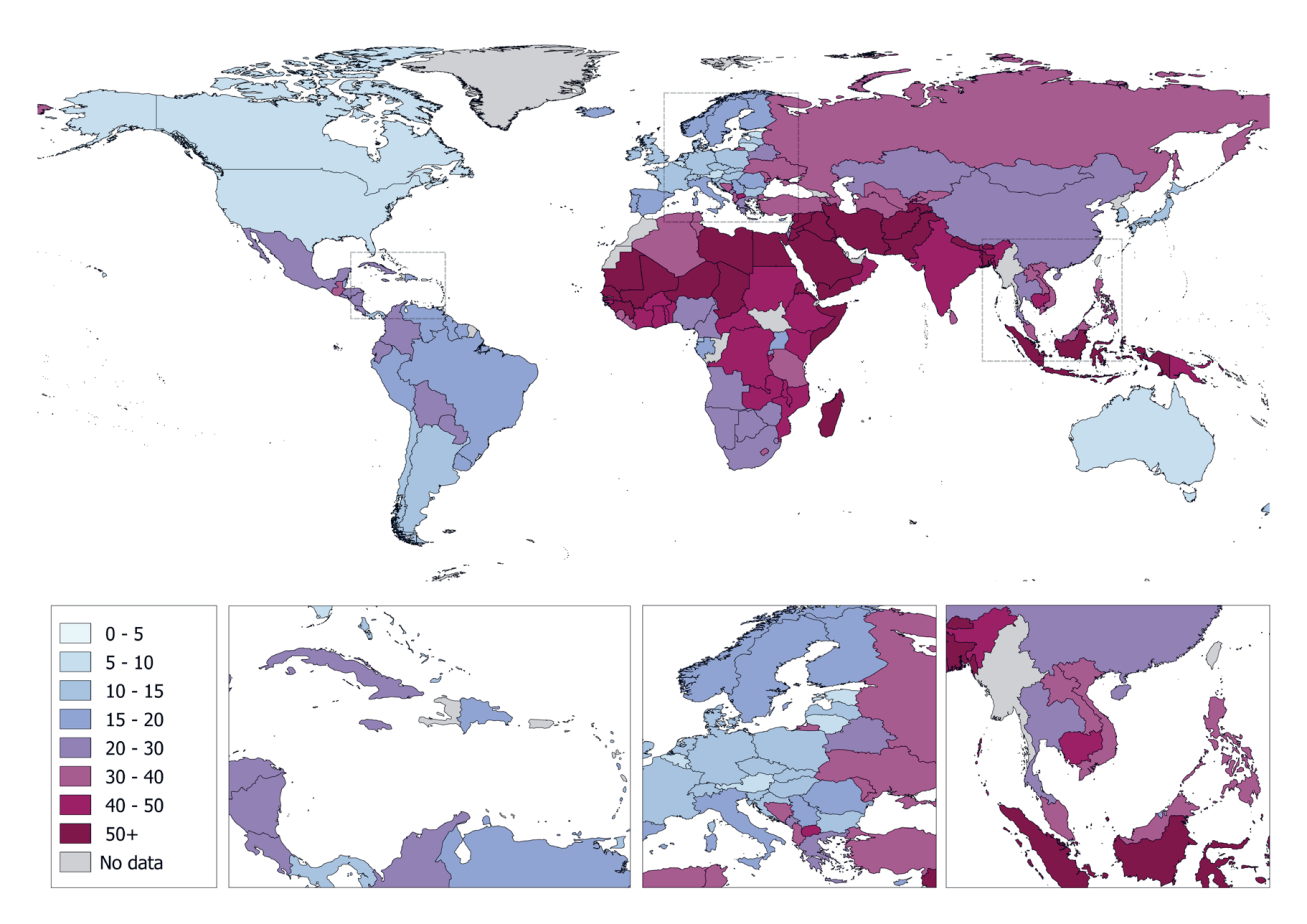
The country-specific proportion (%) of unrecorded alcohol consumption of the total alcohol consumption per capita among adults in 2015.

**Figure 2:**
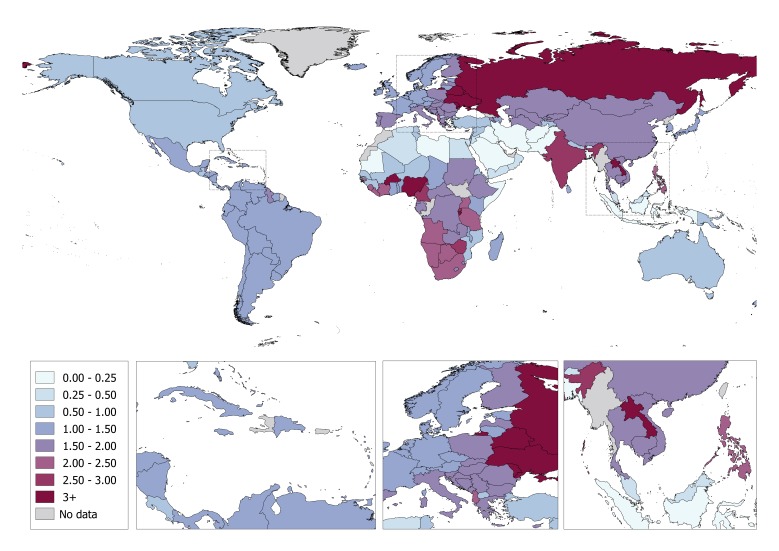
The country-specific volume of unrecorded alcohol consumed per capita in 2015 (litres of pure alcohol).

At the country level, Yemen and Somalia had the highest proportion of unrecorded alcohol consumption (88% and 86% of total alcohol consumed respectively) (**Figure 1**; Table S4 in **Online Supplementary Document**), while the Republic of Moldova and Ukraine had the highest volume of unrecorded alcohol consumption (5.5 and 5.0 L *per capita,* respectively) (**Figure 2**; Table S4 in **Online Supplementary Document**). Conversely, the lowest proportion of unrecorded alcohol consumption was observed for the Americas (14% of total alcohol consumption), while the volume of unrecorded alcohol consumed was lowest in the Eastern Mediterranean (0.3 L *per capita*). The proportion of unrecorded alcohol was also associated with the income of a country, ranging from 15% in high-income countries to 42% in lower-middle-income countries. The volume of unrecorded alcohol consumed was similar across income groups (around 1.5 L *per capita*), with the exception being lower-middle-income countries where unrecorded alcohol consumption was 1.9 L *per capita*. Table S4 in **Online Supplementary Document** shows the country-specific modelled estimates of the proportion (in %) and volume (in litres) of unrecorded and total APC consumed in 2015, as well as the proportion of unrecorded APC consumed based on other data sources.

**Figure 3:**
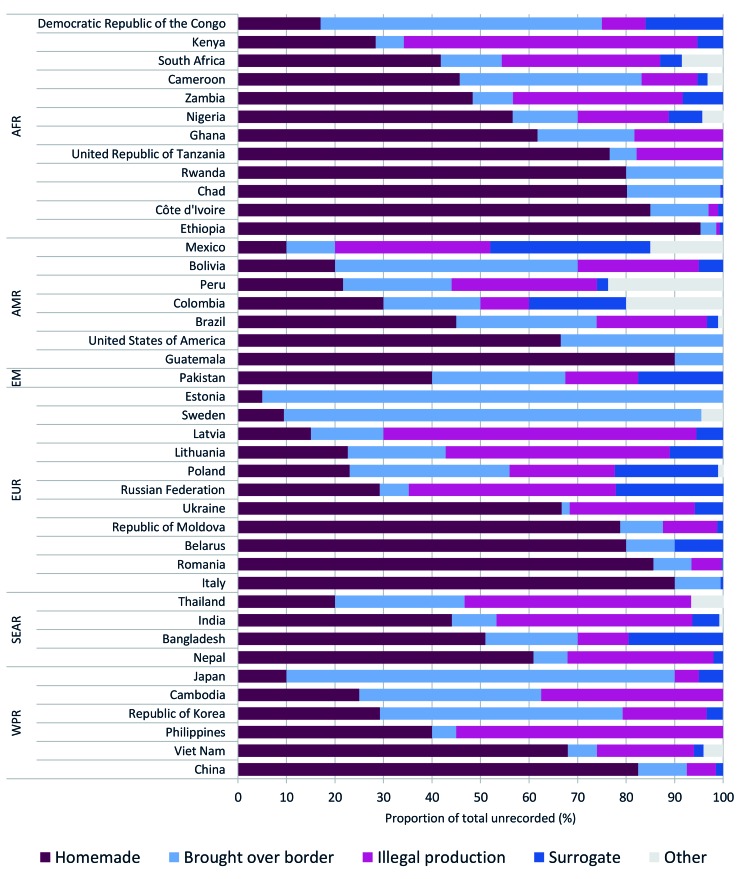
Sources of unrecorded alcohol in 2015 based on the nominal group expert assessment, by World Health Organization region, AFR – African Region, AMR – Region of the Americas, EM – Eastern Mediterranean Region, EUR – European Region, SEAR – South East Asian Region, WPR – Western Pacific Region.

The sources of unrecorded alcohol in 41 countries are outlined in **Figure 3**. In 18 of the 41 countries, homemade alcohol was the source of over one-half of the unrecorded alcohol consumed; however, the relative importance of the sources varied by country, with homemade alcohol accounting for less than 10% of all unrecorded alcohol consumed in Japan, Sweden, and Estonia. In these three countries, cross-border shopping was responsible for 80% or more of the unrecorded alcohol consumed. Illegal production was also common in many countries, accounting for 40% to 65% of the unrecorded alcohol consumed in Eastern Europe (Latvia, Lithuania, and Russia), the Philippines, India, Kenya, and Thailand. Surrogate alcohol was the least common source of unrecorded alcohol, accounting for less than 5% of the total unrecorded alcohol consumed in most countries; however, in Bangladesh, Colombia, Mexico, Poland, and Russia, surrogate alcohol accounted for 20% to 33% of the unrecorded alcohol consumed.

### Perceptions of unrecorded alcohol consumption as a problem

The majority of experts considered unrecorded alcohol consumption to be a public health (62%), social (60%), and financial problem (54%); however, regional differences in these perceptions existed. Logistic regressions showed that compared to high-income countries, experts from low- (odds ratio (OR) 3.87, 95% confidence interval (CI) 1.17-14.57) and lower-middle-income countries (OR = 5.54, 95% CI = 2.05-16.02) were more likely to perceive unrecorded alcohol as a public health problem. Experts from Africa (OR = 5.20, 95% CI = 1.71-16.86) and Eastern Europe (OR = 4.77, 95% CI = 1.13-25.51) were more likely to perceive unrecorded alcohol consumption as a public health problem when compared to experts from Europe (exclusive of Eastern Europe). Experts from the Western Pacific were less likely to perceive unrecorded alcohol as a social problem (OR = 0.19, 95% CI = 0.04-0.80). No other statistically significant differences in the perception of unrecorded alcohol as a problem across regions were observed.

## DISCUSSION

This study found that unrecorded alcohol consumption is high globally, and constitutes a substantial proportion of the total alcohol consumption *per capita* among adults. There was considerable variation between WHO regions with respect to the proportion of unrecorded alcohol (ranging from 20% in the Region of the Americas to 57% in the Eastern Mediterranean Region) as well as the volume of unrecorded alcohol (ranging from 0.3 L in the Eastern Mediterranean Region to 2.1 L in the European Region). The majority of experts, particularly from low- and lower-middle-income countries, and from Africa and Eastern Europe, perceived unrecorded alcohol consumption as a public health problem.

Reduction of taxes on recorded alcohol is often suggested as a method to reduce unrecorded alcohol consumption; however, from a public health perspective, lowering taxes on recorded consumption is likely to increase total alcohol consumption and related harms [40,41]. Specific policy measures aimed at decreasing unrecorded alcohol consumption depend upon the source(s) of the unrecorded alcohol. First, in countries such as Russia and Poland where surrogate alcohol is commonly consumed, taxing surrogates, reducing container sizes, and/or treating these products with bittering agents have been suggested as effective interventions [13,42,43]. Furthermore, enforcing bans on toxic ingredients, such as methanol, in products commonly used as surrogate alcohol, may reduce the harms caused by their consumption [43]. Second, in countries such as Sweden, where border trade is the most important source of unrecorded alcohol, implementing or increasing import fees and stricter border controls could be advisable [43,44]; however, such measures may violate trade agreements (such as the free trade laws of the European Union) [45]. Third, in countries where (large scale) illegal production is highly prevalent, such as in Latvia, Lithuania, and Russia, tax stamps could be used to improve the monitoring and enforcement of policies to reduce consumption. Lastly, for homemade alcohol, the main source of unrecorded alcohol in most countries, policy measures addressing production and consumption are limited, as in many cases the product is not illegal and/or is part of cultural traditions. In these countries, incentivizing registration and quality control of home production have been suggested to improve monitoring and reduce potential harms [43,46]. Given the current lack of international evidence on the effectiveness of policies aimed at reducing the consumption of unrecorded alcohol, there is a critical need to investigate in different global settings the effectiveness of policies and interventions aimed at reducing such consumption and its resulting harms.

Many countries (in particular poorer countries and those undergoing transitions) do not conduct regular population health surveys, nor do they have the health information systems required to capture data on alcohol consumption and alcohol-related harms [2]. Furthermore, many countries lack data on alcohol consumption differentiated by factors such as socioeconomic status; people with a low socioeconomic status have worse health outcomes related to alcohol consumption [47,48] and are more likely to consume surrogate alcohols [49]. Therefore, there is a need for expanded and improved risk factor surveillance systems, including unrecorded alcohol use in particular. These data are necessary to plan, implement, and evaluate prevention and control policies concerning harmful alcohol consumption [4,50].

### Limitations

Any statistical prediction is a model, which is imperfect and relies on many factors, most importantly on the underlying theory and relationships of dependent and independent variables, and the availability and measurement of these variables. There are limitations that are inherent in the estimation of unrecorded alcohol consumption; although the observed outcomes were based on the best available data, the estimates of unrecorded alcohol consumption should be interpreted cautiously, especially for countries where estimations are based on insufficient, and/or potentially biased data. For example, in countries with overall low alcohol consumption (especially for countries, where alcohol is prohibited), small absolute changes in recorded consumption will result in marked changes in the proportion of unrecorded alcohol; however, such countries make relatively small contributions to the global alcohol burden of disease [2,51]. This study also found that unrecorded alcohol consumption as measured by the STEPs surveys was significantly lower than expert estimates, which may indicate a reluctance to disclose unrecorded alcohol use in surveys. To address this, the regressions used to estimate unrecorded alcohol consumption used the nominal group method as the reference method (see Appendix S3 in **Online Supplementary Document**).

## CONCLUSION

This study outlines the most up-to-date and comprehensive estimates of country level unrecorded alcohol consumption, by source. While surveillance of unrecorded alcohol use and the resulting harms ideally involves a direct measurement, this study represents an important step towards transparent and more accurate estimates of unrecorded alcohol consumption. Furthermore, given the variability of the volumes of unrecorded alcohol consumption, this study supports the need for the development and implementation of country-specific policies and programs to reduce alcohol consumption and the resulting harms.

## Additional material

Online Supplementary Document
