# Supplementary material for: The global proportion and volume of unrecorded alcohol in 2015
Source: J Glob Health. 2019 Feb 9;9(1):010421. doi: 10.7189/jogh.09.010421 (PMC6513411; doi:10.7189/jogh.09.010421)
Supplement: Online Supplementary Document [file jogh-09-010421-s001.pdf]

J Glob Health 2019; 9: 010421

# SUPPLEMENTARY INFORMATION

S1 Table. Survey year, response rate in percent, sample size, and number of current drinkers in STEPS surveys ..... 2

S2 Text. Supplementary information on the methodology ..... 3

S3 Text. Questionnaire used in nominal group expert assessment..... 4

S4 Table. Detailed findings by country: Proportion of unrecorded alcohol *per capita* in 2015 as per World Health Organization (WHO) estimates, nominal group expert assessment, the STEPwise approach to surveillance surveys, and the prediction model. Volume of unrecorded and total alcohol consumption *per capita* in litres of pure alcohol were based on the estimated proportion of unrecorded alcohol consumed and the litres of recorded alcohol consumed reported by the WHO ..... 14

S5 Table. Guidelines for Accurate and Transparent Health Estimates Reporting (GATHER) checklist..... 23

**S1 Table. Survey year, response rate in percent, sample size, and number of current drinkers in STEPS surveys**

| Country | Survey year | Response rate (%) | Sample size | N current drinkers |
|---|---|---|---|---|
| Burkina Faso | 2013 | 99 | 4692 | 1114 |
| Bhutan | 2014 | 97 | 2819 | 928 |
| Botswana | 2014 | n.a. | 4065 | 633 |
| Kenya | 2015 | 87 | 4498 | 622 |
| Kyrgyzstan | 2013 | n.a. | 2622 | 473 |
| Kuwait | 2014 | 89 | 3915 | 17 |
| Sri Lanka | 2015 | 72 | 5171 | 469 |
| Republic of Moldova | 2013 | 84 | 4768 | 2057 |
| Myanmar | 2014 | 94 | 8268 | 793 |
| Mongolia | 2013 | 98 | 6013 | 849 |
| Swaziland | 2014 | 76 | 3280 | 329 |
| Tokelau | 2014 | n.a. | 554 | 224 |
| Turkmenistan | 2013 | n.a. | 5110 | 319 |
| Timor-Leste | 2014 | 96 | 2601 | 336 |
| Uganda | 2014 | 99 | 3982 | 930 |
| Uzbekistan | 2014 | 89 | 3830 | 358 |

## S2 Text. Supplementary information on the methodology

### Assessment of unrecorded alcohol consumption based on STEPS survey data

Total alcohol consumption was assessed in the WHO's STEPwise approach to surveillance (STEPS) surveys using the following question: "During each of the past 7 days, how many standard drinks did you have each day?" Unrecorded alcohol use was assessed in five categories: "During the past 7 days, did you consume any homebrewed alcohol, any alcohol brought over the border from another country, any alcohol not intended for drinking or other untaxed alcohol?" For each category, the number of standard drinks was assessed: "On average, how many standard drinks of the following did you consume during the past 7 days?" The estimates therefore relied on the assumption that the levels of underreporting of unrecorded and total alcohol use were approximately the same.

### Description of obtaining expert judgments on unrecorded consumption

To date, only experts' judgments of unrecorded alcohol consumption have been available for the majority of countries. Various procedures were used for collecting data on unrecorded alcohol consumption: up to and including 2010, experts were identified as general experts on alcohol consumption, often advising on regions and/or more than one country. The collection of expert judgments became more standardized over time, and the following procedures had been implemented since 2010.

### Changes Implemented after 2010

A written questionnaire (see below) was sent to experts from the country being considered, requesting their assessment of estimates of unrecorded alcohol consumption and of supporting documentation. A limited number of countries were selected based on the following criteria: the proportion of unrecorded alcohol consumption based on prior estimates, the size of the country, and any known controversies surrounding the estimates of unrecorded alcohol consumption contained in the Global Status Report on Alcohol and Health.[43] The countries selected covered 63% of the world's population in 2010 (for a more detailed description, see[13]). For countries where more than one expert's opinion was solicited, a second round of questioning followed: based on all information obtained in the first round of questioning, experts were asked again for their final estimates of unrecorded alcohol consumption, thereby applying a nominal group technique procedure.[13]

For all other countries, there was at least one expert from each country who was asked for their assessment. The expert opinion survey was anonymously completed. As the experts were country-specific after 2010, and they often provided documentation to support their judgments, albeit often pertaining to certain sources of unrecorded consumption or non-nationally representative information, we used these expert judgements in cases where there were changes from the original 2014 Global Status Report on Alcohol and Health estimates.

While the questionnaire asked about the proportion of unrecorded alcohol consumed in total alcohol *per capita* consumption, rather than the absolute level of unrecorded consumption (in litres of pure alcohol), some experts only answered with absolute levels. Using recorded consumption estimates these estimates were transformed into the percentage of overall consumption.

**S3 Text. Questionnaire used in nominal group expert assessment**

| **Date:** | | | 2015 | *(Day/Month/Year)* |
|---|---|---|---|---|

| **Country:** | **[*country name here*]** |
|---|---|

*Questionnaire completed by:*

| **Last name:** | | **First name:** | |
|---|---|---|---|

| **Title/Position:** | |
|---|---|

| **Institute/Organization/Etc:** | |
|---|---|
| | |

| | |
|---|---|
| **Phone:** | |
| **Fax:** | |
| **e-mail:** | |

**Contact details for questions or clarifications:**

***World Health Organization***
Management of Substance Abuse
Department of Mental Health and Substance Abuse
World Health Organization
20, Avenue Appia
CH-1211 Geneva 27
Switzerland
Phone: +41 22 791 33 34; Fax: +41 22 791 48 51; e-mail: gisah@who.int

***Centre for Addiction and Mental Health***
Social and Epidemiology Research Department, PAHO/WHO Collaborating Centre for Addiction and Mental Health
Centre for Addictions and Mental Health (CAMH)
33 Russell Street
Toronto, Ontario, Canada, M5S 2S1
Phone: +1 416 535 8501 ext. 36173; Fax: +1 416 595 6033; e-mail: jurgen.rehm@CAMH.ca

## THE QUESTIONNAIRE ON UNRECORDED ALCOHOL CONSUMPTION

**Dear Expert,**

**We invite you to participate in this additional component of the WHO Global survey on alcohol and health in your personal capacity with an objective to improve our understanding of a number of critical issues related to unrecorded alcohol consumption in different parts of the world.**

**Any alcohol that is consumed as an alcoholic beverage, but not taxed or registered as such under the jurisdiction of where it is consumed, is regarded as unrecorded alcohol. Unrecorded alcohol in a country includes homemade or informally produced alcohol (legal or illegal), illegally produced (on a large scale) alcohol outside the usual system of governmental control, smuggled alcohol, alcohol obtained through cross-border shopping, and alcohol consumed as a substitute of alcoholic beverages, but not intended for such consumption (industrial ethanol, ethanol-containing liquids, ethanol-based medicines, etc.**

**Please note that we are interested in your responses as they pertain to the national perspective. If your responses pertain to a sub-national or regional perspective, please indicate below to which sub-national population or region your responses pertain.**

*(Please type directly into the text box)*

**Q1. Currently we have an estimate[1] that [*country specific estimate here*]% of all the alcohol that is consumed in [*country name here*] is unrecorded. This corresponds to [*country specific estimate here*] litres of adult (15+ years old) per capita per year (for the year of 2015)[2]. The estimated total adult per capita consumption is [*country specific estimate here*] litres of pure alcohol. Do you agree with this estimate of unrecorded alcohol consumption?**

☐**Yes** ☐ **No**

**How certain are you about your answer to Q1? (1-5)**

*(Please tick, highlight or circle the appropriate option)*

| | |
|---|---|
| ☐(1) Very certain | (99-80% confidence of being right) |
| ☐(2) Quite certain | (79-60% confidence of being right) |
| ☐(3) Not so certain | (59-40% confidence of being right, i.e. either way) |
| ☐(4) Quite uncertain | (39-20% confidence of being right) |
| ☐(5) Very uncertain | (less than 20% confidence of being right) |

**If the answer to Question 1 is "Yes", please proceed to Q3.**

**If the answer to Question 1 is "No", please proceed to Q2.**

**Please provide any source or any form of evidence in relation to your response to this question (Q1); sources could be official webpages, government reports, statistics from customs, research articles etc.:**

*(Please type directly into the text box)*

[1]The presented estimate is based on the best available data in WHO from different sources including Food and Agricultural Organization (FAO), industry, expert opinions and research.

[2]Please focus on proportion of unrecorded alcohol consumption from the total and not on the absolute figures of per capita consumption. If you have different opinion on the total per capita consumption in [*country name here*] for 2015 please provide your views in Q2, Q4 and Q8. We have special procedures for validating the total per capita alcohol consumption for 2015.

**Q2. What is your estimation of unrecorded alcohol consumption in [*country name here*]? (please give an answer even if it is with very high level of uncertainty):**
**___% of all alcohol consumed in the country, which corresponds to ___litres of adult (15+ years old) per capita unrecorded alcohol consumption per year (for the year 2015).**

**How certain are you about your answer to Q2? (1-5)**
*(Please tick, highlight or circle the appropriate option)*

| | |
|---|---|
| ☐(1) Very certain | (99-80% confidence of being right) |
| ☐(2) Quite certain | (79-60% confidence of being right) |
| ☐(3) Not so certain | (59-40% confidence of being right, i.e. either way) |
| ☐(4) Quite uncertain | (39-20% confidence of being right) |
| ☐(5) Very uncertain | (less than 20% confidence of being right) |

**Please provide any source or any form of evidence in relation to your response to this question (Q2); sources could be official webpages, government reports, statistics from customs, research articles etc.:**

*(Please type directly into the text box)*

**Q3. Considering the unrecorded alcohol only, please provide us with your expert opinion on % estimates for the main categories of unrecorded alcohol (adding up to the total, i.e. 100% of unrecorded alcohol) consumed in [*country name here*]:**
*(Please type directly into the parentheses)*

**Home produced alcohol** **( ) % from total unrecorded**

- Home produced spirits ( ) % from total unrecorded
- Home produced beer ( ) % from total unrecorded
- Home produced wine ( ) % from total unrecorded

**Brought over the border** **( ) % from total unrecorded**

- Smuggling, contraband (large scale) ( ) **%** from total unrecorded
- Duty-free and cross-border shopping ( ) **%** from total unrecorded

**Illegal production** **( ) % from total unrecorded**

**Surrogate alcohol, industrial spirits not intended for human consumption**
**( ) % from total unrecorded**

**Other (specify):**

**( ) % from total unrecorded**

**How certain are you about your answers to Q3? (1-5)**
*(Please tick, highlight or circle the appropriate option)*

☐(1) Very certain (99-80% confidence of being right)
☐(2) Quite certain (79-60% confidence of being right)
☐(3) Not so certain (59-40% confidence of being right, i.e. either way)
☐(4) Quite uncertain (39-20% confidence of being right)
☐(5) Very uncertain (less than 20% confidence of being right)

**Please provide any source or any form of evidence in relation to your responses to this question (Q3); sources could be official webpages, government reports, statistics from customs, research articles etc.:**
*(Please type directly into the text box)*

**Q4. Is unrecorded alcohol consumption perceived as a matter that demands attention in [*country name here*]?**

☐**YES, please GO TO questions 4a – 4c.**
☐**NO, please GO TO question 5.**

**4a. Is the consumption of unrecorded alcohol perceived as a financial problem, for example, due to loss of taxes?**

☐**Yes** ☐**No**

**4b. Is the consumption of unrecorded alcohol perceived as a public health problem, for example, due to outbreaks of methanol intoxication or implied high toxicity of unrecorded alcohol?**

☐**Yes** ☐**No**

**4c. Is the consumption of unrecorded alcohol perceived as a social problem, for example, due to smuggling or illegal large scale production?**

☐**Yes** ☐**No**

**How certain are you about your answer to Q4a-c? (1-5)**
*(Please tick, highlight or circle the appropriate option)*

| | |
|---|---|
| ☐(1) Very certain | (99-80% confidence of being right) |
| ☐(2) Quite certain | (79-60% confidence of being right) |
| ☐(3) Not so certain | (59-40% confidence of being right, i.e. either way) |
| ☐(4) Quite uncertain | (39-20% confidence of being right) |
| ☐(5) Very uncertain | (less than 20% confidence of being right) |

**Please provide any source or any form of evidence in relation to your responses to this question (Q4); sources could be official webpages, excerpts from national strategies and governmental reports, statistics from customs, research articles etc.:**
*(Please type directly into the text box)*

**Q5. Please describe the social and demographic profile of the majority of consumers of unrecorded alcohol (e.g. race, sex, age, socioeconomic status, state of mental health, addiction disorders)?**

**Home produced alcohol:**

**5a. Home produced alcohol:**

*(Please type directly into the text box)*

**5b. Surrogate alcohol, industrial spirits not intended for human consumption:**

*(Please type directly into the text box)*

**5c. Other category of unrecorded alcohol (specify)**

*(Please type directly into the text box)*

**Q6. Please list the methods that are currently used in [*country name here*] to deter people from making or consuming unrecorded alcohol (Please tick, highlight or circle the appropriate option and provide additional information as appropriate).**
*(Please tick, highlight or circle the appropriate option and provide additional information as appropriate)*

☐Using tax stamps on alcohol beverages

☐Reducing taxes on recorded alcohol

☐Strengthening legislation and law enforcement measures

☐Providing financial incentives to home-producers and others to encourage quality control and registration

☐ Adding bittering agents to surrogate alcohols

☐Issuing public warnings about contaminants and other health threats from informal or illicit alcohol

☐Reducing sizes of ethanol-containing medicines

☐Allowing sales of ethanol-containing medicines only by prescription

☐Other, please specify:

*(Please type directly into the text box)*

**articles etc.**
*(Please type directly into the text box)*

**Q7. Did we miss any aspect of unrecorded alcohol? Please specify.**

*(Please type directly into the text box)*

**Q8. Please recommend the names and contact e-mail or telephone numbers information of at least 2 other expert(s) on unrecorded alcohol in [*country name here*] that we should contact on this issue?**

| | |
|---|---|
| **1.** | |
| | |
| **2.** | |
| | |
| **3.** | |
| | |
| **4.** | |
| | |

**We truly appreciate your time and cooperation. Thank you.**

**S4 Table. Detailed findings by country: Proportion of unrecorded alcohol *per capita* in 2015 as per World Health Organization (WHO) estimates, nominal group expert assessment, the STEPwise approach to surveillance surveys, and the prediction model. Volume of unrecorded and total alcohol consumption *per capita* in litres of pure alcohol were based on the estimated proportion of unrecorded alcohol consumed and the litres of recorded alcohol consumed reported by the WHO**

| | | | Proportion unrecorded alcohol based on other data sources (%) | | | Proportion unrecorded alcohol based on the statistical model (%) | | | Volume of alcohol *per capita* based on the statistical model (litres of pure alcohol ) | | | |
|---|---|---|---|---|---|---|---|---|---|---|---|---|
| WHO region | Income level | Country | WHO | Experts | STEPS | % unrecorded | 95% confidence interval | | Unrecorded alcohol | 95% confidence interval | | Total alcohol |
| AFR | UMIC | Algeria | 39.4 | | | 40.0 | 24.0 | 41.8 | 0.4 | 0.2 | 0.4 | 1.0 |
| | UMIC | Angola | 18.0 | | | 21.6 | 16.1 | 26.4 | 2.4 | 1.7 | 3.2 | 11.3 |
| | LIC | Benin | 44.1 | | | 48.9 | 40.8 | 54.8 | 1.4 | 1.0 | 1.7 | 2.8 |
| | UMIC | Botswana | 33.4 | | 11.8 | 26.5 | 23.8 | 36.4 | 2.1 | 1.8 | 3.3 | 7.8 |
| | LIC | Burkina Faso | 39.0 | | 32.5 | 40.4 | 38.5 | 52.6 | 3.2 | 2.9 | 5.1 | 7.8 |
| | LIC | Burundi | 42.0 | | | 44.9 | 37.0 | 52.7 | 3.3 | 2.3 | 4.5 | 7.3 |
| | LMIC | Cameroon | 30.0 | 60.0 | | 26.9 | 14.0 | 39.9 | 2.6 | 1.1 | 4.6 | 9.5 |
| | LMIC | Cape Verde | 37.9 | | | 28.3 | 27.4 | 40.5 | 2.0 | 1.9 | 3.4 | 7.1 |
| | LIC | Central African Republic | 55.1 | | | 49.1 | 46.2 | 60.2 | 1.6 | 1.5 | 2.6 | 3.4 |
| | LIC | Chad | 77.1 | 43.5 | | 55.5 | 42.5 | 68.5 | 1.5 | 0.9 | 2.6 | 2.7 |
| | LIC | Comoros | 44.4 | | | 84.5 | 54.9 | 80.6 | 0.5 | 0.1 | 0.4 | 0.6 |
| | LMIC | Congo | 38.0 | | | | | | | | | |
| | LMIC | Côte d'Ivoire | 39.0 | 32.0 | | 42.7 | 31.6 | 46.3 | 2.3 | 1.5 | 2.7 | 5.5 |
| | LIC | Democratic Republic of the Congo | 39.9 | 55.3 | | 49.0 | 40.3 | 57.6 | 1.7 | 1.2 | 2.5 | 3.6 |
| | HIC | Equatorial Guinea | 7.5 | | | 12.7 | 8.1 | 15.9 | 1.6 | 0.9 | 2.0 | 12.3 |
| | LIC | Eritrea | 50.0 | | | 55.6 | 36.6 | 53.2 | 0.8 | 0.4 | 0.7 | 1.4 |
| | LIC | Ethiopia | 58.1 | 58.0 | | 49.8 | 49.3 | 64.0 | 1.9 | 1.9 | 3.4 | 3.8 |
| | UMIC | Gabon | 18.0 | | | 16.7 | 13.3 | 23.1 | 1.8 | 1.4 | 2.7 | 10.6 |

| | | | Proportion unrecorded alcohol based on other data sources (%) | | | Proportion unrecorded alcohol based on the statistical model (%) | | | Volume of alcohol *per capita* based on the statistical model (litres of pure alcohol ) | | | |
|---|---|---|---|---|---|---|---|---|---|---|---|---|
| **WHO region** | **Income level** | **Country** | **WHO** | **Experts** | **STEPS** | **% unrecorded** | **95% confidence interval** | | **Unrecorded alcohol** | **95% confidence interval** | | **Total alcohol** |
| | LIC | Gambia | 24.9 | | | | | | | | | |
| | LMIC | Ghana | 63.0 | 59.0 | | 41.4 | 49.3 | 63.8 | 1.2 | 1.6 | 2.9 | 2.8 |
| | LIC | Guinea | 71.1 | | | 69.9 | 63.1 | 78.4 | 0.5 | 0.4 | 0.8 | 0.7 |
| | LIC | Guinea-Bissau | 33.4 | | | 40.8 | 32.6 | 44.4 | 2.5 | 1.7 | 2.9 | 6.0 |
| | LMIC | Kenya | 58.0 | 57.5 | 20.2 | 44.6 | 45.8 | 62.2 | 1.5 | 1.6 | 3.0 | 3.3 |
| | LMIC | Lesotho | 58.1 | | | 39.9 | 43.2 | 56.9 | 1.6 | 1.8 | 3.2 | 4.0 |
| | LIC | Liberia | 30.9 | | | 39.0 | 30.7 | 42.1 | 2.4 | 1.6 | 2.7 | 6.1 |
| | LIC | Madagascar | 50.0 | | | 54.3 | 36.2 | 52.3 | 1.0 | 0.5 | 1.0 | 1.9 |
| | LIC | Malawi | 48.1 | | | 45.3 | 40.7 | 55.1 | 1.0 | 0.8 | 1.5 | 2.2 |
| | LIC | Mali | 45.8 | | | 53.8 | 34.3 | 49.8 | 0.8 | 0.3 | 0.6 | 1.4 |
| | LMIC | Mauritania | 90.9 | | | 84.1 | 78.0 | 92.3 | 0.1 | 0.0 | 0.1 | 0.1 |
| | UMIC | Mauritius | 25.0 | | | 31.0 | 23.1 | 36.1 | 1.4 | 0.9 | 1.7 | 4.4 |
| | LIC | Mozambique | 50.0 | | | 46.2 | 42.2 | 56.4 | 1.0 | 0.8 | 1.5 | 2.2 |
| | UMIC | Namibia | 25.0 | | | 20.7 | 18.0 | 29.1 | 2.3 | 2.0 | 3.6 | 11.2 |
| | LIC | Niger | 66.7 | | | 78.9 | 64.1 | 83.0 | 0.6 | 0.3 | 0.7 | 0.7 |
| | LMIC | Nigeria | 15.0 | 16.2 | | 28.1 | 13.8 | 22.9 | 3.0 | 1.2 | 2.3 | 10.8 |
| | LIC | Rwanda | 27.5 | 30.5 | | 23.2 | 21.5 | 33.8 | 2.5 | 2.3 | 4.3 | 10.9 |
| | LMIC | Sao Tome and Principe | 35.0 | | | 29.2 | 27.1 | 39.0 | 2.3 | 2.1 | 3.6 | 8.0 |
| | LMIC | Senegal | 50.0 | | | 67.6 | 51.0 | 69.1 | 0.5 | 0.3 | 0.6 | 0.8 |
| | HIC | Seychelles | 17.5 | | | 14.0 | 11.3 | 21.1 | 1.6 | 1.2 | 2.6 | 11.3 |
| | LIC | Sierra Leone | 34.0 | | | 39.5 | 32.3 | 44.0 | 2.5 | 1.8 | 3.0 | 6.2 |
| | UMIC | South Africa | 37.0 | 21.3 | | 23.3 | 18.7 | 30.3 | 2.1 | 1.6 | 3.1 | 9.2 |
| | LIC | South Sudan | | | | | | | | | | |
| | LMIC | Swaziland | 17.1 | | 13.9 | 27.0 | 19.7 | 30.9 | 1.9 | 1.3 | 2.3 | 7.1 |
| | LIC | Togo | 45.0 | | | 46.3 | 40.1 | 53.8 | 1.2 | 1.0 | 1.7 | 2.7 |

| WHO region | Income level | Country | Proportion unrecorded alcohol based on other data sources (%) | | | Proportion unrecorded alcohol based on the statistical model (%) | | | Volume of alcohol *per capita* based on the statistical model (litres of pure alcohol ) | | | |
|---|---|---|---|---|---|---|---|---|---|---|---|---|
| | | | WHO | Experts | STEPS | % unrecorded | 95% confidence interval | | Unrecorded alcohol | 95% confidence interval | | Total alcohol |
| | LIC | Uganda | 13.0 | | 42.3 | 19.4 | 22.7 | 37.6 | 2.5 | 3.0 | 6.2 | 12.7 |
| | LIC | United Republic of Tanzania | 33.4 | 48.0 | | 34.4 | 38.0 | 50.7 | 2.2 | 2.6 | 4.3 | 6.4 |
| | LMIC | Zambia | 38.0 | 41.2 | | 40.4 | 34.5 | 47.5 | 1.6 | 1.3 | 2.2 | 4.0 |
| | LIC | Zimbabwe | 16.0 | | | 26.1 | 18.0 | 28.0 | 2.5 | 1.6 | 2.8 | 9.7 |
| AMR | HIC | Antigua and Barbuda | 5.0 | | | 12.0 | 7.6 | 13.8 | 1.2 | 0.7 | 1.4 | 9.8 |
| | HIC | Argentina | 11.0 | | | 13.8 | 10.0 | 17.5 | 1.3 | 0.9 | 1.7 | 9.4 |
| | HIC | Bahamas | 4.9 | | | 12.1 | 7.6 | 13.9 | 1.2 | 0.7 | 1.5 | 10.3 |
| | HIC | Barbados | 5.5 | | | 13.0 | 8.2 | 14.8 | 1.3 | 0.8 | 1.5 | 10.1 |
| | UMIC | Belize | 20.0 | | | 18.8 | 15.4 | 25.2 | 1.5 | 1.2 | 2.2 | 8.1 |
| | LMIC | Bolivia (Plurinational State of) | 35.0 | 27.2 | | 25.1 | 23.3 | 36.2 | 1.3 | 1.2 | 2.2 | 5.1 |
| | UMIC | Brazil | 16.7 | 24.0 | | 15.9 | 16.5 | 27.5 | 1.4 | 1.5 | 2.8 | 8.8 |
| | HIC | Canada | 19.2 | | | 9.9 | 9.0 | 17.9 | 0.9 | 0.8 | 1.8 | 9.0 |
| | HIC | Chile | 20.5 | | | 14.8 | 13.1 | 22.4 | 1.2 | 1.1 | 2.1 | 8.4 |
| | UMIC | Colombia | 15.0 | 14.5 | | 23.6 | 14.4 | 24.6 | 1.4 | 0.7 | 1.4 | 5.7 |
| | UMIC | Costa Rica | 21.6 | | | 23.7 | 18.2 | 29.9 | 1.0 | 0.7 | 1.4 | 4.2 |
| | UMIC | Cuba | 19.5 | | | 24.0 | 17.4 | 29.3 | 1.4 | 0.9 | 1.8 | 5.7 |
| | UMIC | Dominica | 6.5 | | | 19.8 | 11.9 | 19.9 | 1.4 | 0.7 | 1.4 | 6.8 |
| | UMIC | Dominican Republic | 10.0 | | | 18.4 | 12.2 | 20.3 | 1.3 | 0.8 | 1.5 | 7.3 |
| | UMIC | Ecuador | 25.0 | | | 24.7 | 20.2 | 31.9 | 1.3 | 1.0 | 1.8 | 5.1 |
| | LMIC | El Salvador | 29.6 | | | 29.5 | 24.0 | 37.8 | 1.0 | 0.8 | 1.5 | 3.4 |
| | UMIC | Grenada | 8.0 | | | 13.9 | 9.2 | 16.4 | 1.4 | 0.9 | 1.7 | 9.8 |
| | LMIC | Guatemala | 43.2 | 52.9 | | 35.8 | 36.0 | 53.5 | 1.0 | 1.0 | 2.0 | 2.8 |

| WHO region | Income level | Country | Proportion unrecorded alcohol based on other data sources (%) WHO | Experts | STEPS | Proportion unrecorded alcohol based on the statistical model (%) % unrecorded | 95% confidence interval | | Volume of alcohol *per capita* based on the statistical model (litres of pure alcohol ) Unrecorded alcohol | 95% confidence interval | | Total alcohol |
|---|---|---|---|---|---|---|---|---|---|---|---|---|
| | LMIC | Guyana | 11.7 | | | 18.4 | 12.4 | 21.5 | 1.7 | 1.1 | 2.1 | 9.4 |
| | LIC | Haiti | 9.4 | | | | | | | | | |
| | LMIC | Honduras | 24.5 | | | 29.4 | 22.1 | 35.0 | 1.2 | 0.8 | 1.5 | 4.1 |
| | UMIC | Jamaica | 30.0 | | | 24.2 | 21.7 | 34.2 | 1.2 | 1.1 | 2.0 | 5.1 |
| | UMIC | Mexico | 25.5 | 39.2 | | 20.5 | 24.3 | 38.0 | 1.4 | 1.7 | 3.2 | 6.6 |
| | LMIC | Nicaragua | 30.8 | | | 26.6 | 23.2 | 36.2 | 1.3 | 1.1 | 2.0 | 4.8 |
| | UMIC | Panama | 10.4 | | | 14.7 | 10.4 | 17.8 | 1.2 | 0.8 | 1.5 | 8.3 |
| | UMIC | Paraguay | 21.0 | | | 22.7 | 17.3 | 29.1 | 1.5 | 1.0 | 2.1 | 6.5 |
| | UMIC | Peru | 35.0 | 24.1 | | 19.3 | 19.8 | 31.2 | 1.4 | 1.4 | 2.6 | 7.1 |
| | HIC | Saint Kitts and Nevis | 5.5 | | | | | | | | | |
| | UMIC | Saint Lucia | 1.9 | | | 13.1 | 7.4 | 13.7 | 1.4 | 0.7 | 1.4 | 10.5 |
| | UMIC | Saint Vincent and the Grenadines | 4.1 | | | 16.1 | 9.5 | 16.4 | 1.4 | 0.8 | 1.4 | 8.7 |
| | UMIC | Suriname | 15.9 | | | 17.0 | 13.2 | 21.9 | 1.4 | 1.0 | 1.9 | 8.1 |
| | HIC | Trinidad and Tobago | 10.0 | | | 13.2 | 9.3 | 16.8 | 1.1 | 0.7 | 1.4 | 8.2 |
| | HIC | United States of America | 5.5 | 5.0 | | 8.7 | 4.8 | 10.6 | 0.8 | 0.4 | 1.0 | 9.7 |
| | HIC | Uruguay | 14.1 | | | 17.5 | 12.9 | 21.5 | 1.2 | 0.9 | 1.6 | 7.0 |
| | HIC | Venezuela (Bolivarian Republic of) | 15.0 | | | 18.8 | 13.7 | 23.1 | 1.4 | 0.9 | 1.8 | 7.4 |
| EMR | LIC | Afghanistan | 98.0 | | | 85.9 | 83.8 | 93.7 | 0.1 | 0.1 | 0.1 | 0.1 |
| | HIC | Bahrain | 5.3 | | | | | | | | | |
| | LMIC | Djibouti | 31.4 | | | | | | | | | |
| | LMIC | Egypt | 47.5 | | | 57.7 | 40.4 | 66.8 | 0.3 | 0.1 | 0.4 | 0.5 |

| | | | Proportion unrecorded alcohol based on other data sources (%) | | | Proportion unrecorded alcohol based on the statistical model (%) | | | Volume of alcohol *per capita* based on the statistical model (litres of pure alcohol ) | | | |
|---|---|---|---|---|---|---|---|---|---|---|---|---|
| **WHO region** | **Income level** | **Country** | **WHO** | **Experts** | **STEPS** | **% unrecorded** | **95% confidence interval** | | **Unrecorded alcohol** | **95% confidence interval** | | **Total alcohol** |
| | UMIC | Iran (Islamic Republic of) | 97.1 | | | 73.6 | 75.4 | 89.2 | 0.1 | 0.1 | 0.2 | 0.1 |
| | UMIC | Iraq | 61.4 | | | 55.0 | 45.9 | 71.4 | 0.2 | 0.1 | 0.4 | 0.4 |
| | UMIC | Jordan | 28.0 | | | 53.4 | 29.4 | 55.9 | 0.4 | 0.1 | 0.5 | 0.8 |
| | HIC | Kuwait | 90.9 | | 44.4 | 58.3 | 58.9 | 82.7 | 0.0 | 0.0 | 0.1 | 0.0 |
| | UMIC | Lebanon | 24.2 | | | | | | | | | |
| | UMIC | Libya | 90.9 | | | 74.3 | 72.8 | 88.0 | 0.0 | 0.0 | 0.0 | 0.0 |
| | LMIC | Morocco | 46.9 | | | | | | | | | |
| | HIC | Oman | 26.5 | | | 48.6 | 26.8 | 52.7 | 0.3 | 0.1 | 0.4 | 0.7 |
| | LMIC | Pakistan | 83.3 | 96.8 | | 85.8 | 83.5 | 93.6 | 0.2 | 0.2 | 0.6 | 0.3 |
| | HIC | Qatar | 34.7 | | | | | | | | | |
| | HIC | Saudi Arabia | 52.6 | | | 64.3 | 46.8 | 71.7 | 0.2 | 0.1 | 0.2 | 0.3 |
| | LIC | Somalia | 100.0 | | | 86.4 | 84.8 | 94.2 | 0.0 | 0.0 | 0.0 | 0.0 |
| | LMIC | Sudan | 35.0 | | | 46.5 | 28.0 | 57.6 | 1.8 | 0.8 | 2.9 | 4.0 |
| | LMIC | Syrian Arab Republic | 21.3 | | | 60.7 | 22.0 | 47.9 | 0.9 | 0.2 | 0.5 | 1.5 |
| | UMIC | Tunisia | 13.7 | | | 31.5 | 15.3 | 36.7 | 0.6 | 0.3 | 0.8 | 2.0 |
| | HIC | United Arab Emirates | 46.9 | | | | | | | | | |
| | LMIC | Yemen | 83.3 | | | 88.2 | 78.7 | 92.5 | 0.3 | 0.1 | 0.5 | 0.3 |
| EUR | UMIC | Albania | 28.0 | | | 32.4 | 24.8 | 38.6 | 2.1 | 1.4 | 2.7 | 6.4 |
| | HIC | Andorra | 7.1 | | | 12.6 | 8.3 | 15.2 | 1.4 | 0.9 | 1.7 | 11.1 |
| | LMIC | Armenia | 27.1 | | | 32.2 | 23.9 | 38.7 | 1.9 | 1.2 | 2.5 | 5.8 |
| | HIC | Austria | 4.6 | | | 8.3 | 5.2 | 11.0 | 1.1 | 0.7 | 1.5 | 13.4 |
| | UMIC | Azerbaijan | 33.1 | | | 34.1 | 27.0 | 43.2 | 1.4 | 1.0 | 2.0 | 4.1 |
| | UMIC | Belarus | 15.0 | 15.0 | | 21.0 | 11.2 | 27.8 | 3.7 | 1.8 | 5.4 | 17.7 |
| | HIC | Belgium | 4.7 | | | 8.9 | 6.0 | 10.9 | 1.2 | 0.8 | 1.5 | 13.8 |

| WHO region | Income level | Country | Proportion unrecorded alcohol based on other data sources (%) | | | Proportion unrecorded alcohol based on the statistical model (%) | | | Volume of alcohol *per capita* based on the statistical model (litres of pure alcohol ) | | | |
|---|---|---|---|---|---|---|---|---|---|---|---|---|
| | | | WHO | Experts | STEPS | % unrecorded | 95% confidence interval | | Unrecorded alcohol | 95% confidence interval | | Total alcohol |
| | UMIC | Bosnia and Herzegovina | 35.0 | | | 31.0 | 26.4 | 42.2 | 1.7 | 1.4 | 2.8 | 5.6 |
| | UMIC | Bulgaria | 9.2 | | | 11.3 | 7.7 | 15.4 | 1.6 | 1.0 | 2.3 | 13.9 |
| | HIC | Croatia | 15.0 | | | 12.3 | 10.0 | 17.8 | 1.7 | 1.4 | 2.6 | 13.8 |
| | HIC | Cyprus | 10.0 | | | 15.8 | 11.3 | 17.8 | 1.7 | 1.1 | 1.9 | 10.5 |
| | HIC | Czech Republic | 9.0 | | | 10.4 | 7.7 | 13.6 | 1.4 | 1.0 | 2.0 | 13.9 |
| | HIC | Denmark | 9.0 | | | 12.3 | 8.4 | 15.8 | 1.3 | 0.8 | 1.7 | 10.5 |
| | HIC | Estonia | 4.5 | 5.0 | | 7.3 | 4.3 | 10.0 | 1.2 | 0.7 | 1.7 | 16.2 |
| | HIC | Finland | 22.1 | | | 15.1 | 14.3 | 22.5 | 1.5 | 1.4 | 2.4 | 10.0 |
| | HIC | France | 4.0 | | | 10.8 | 7.1 | 12.1 | 1.4 | 0.9 | 1.6 | 12.6 |
| | LMIC | Georgia | 24.0 | | | | | | | | | |
| | HIC | Germany | 5.0 | | | 10.6 | 7.1 | 12.3 | 1.3 | 0.8 | 1.5 | 12.2 |
| | HIC | Greece | 18.5 | | | 20.7 | 16.4 | 25.2 | 1.8 | 1.4 | 2.3 | 8.8 |
| | HIC | Hungary | 16.3 | | | 13.5 | 11.0 | 19.5 | 1.6 | 1.3 | 2.5 | 11.9 |
| | HIC | Iceland | 5.5 | | | 16.1 | 10.0 | 16.7 | 1.4 | 0.8 | 1.5 | 8.9 |
| | HIC | Ireland | 4.7 | | | 10.7 | 6.9 | 12.6 | 1.2 | 0.7 | 1.4 | 11.3 |
| | HIC | Israel | 10.2 | | | 29.7 | 16.5 | 29.0 | 1.2 | 0.5 | 1.1 | 3.9 |
| | HIC | Italy | 5.0 | 7.0 | | 18.3 | 8.9 | 14.4 | 1.6 | 0.7 | 1.2 | 8.9 |
| | UMIC | Kazakhstan | 30.0 | | | 23.8 | 22.1 | 33.1 | 1.9 | 1.7 | 3.0 | 8.0 |
| | LMIC | Kyrgyzstan | 40.0 | | 8.2 | 35.6 | 27.0 | 43.7 | 1.8 | 1.2 | 2.5 | 5.1 |
| | HIC | Latvia | 15.5 | 14.8 | | 14.4 | 11.7 | 19.0 | 1.7 | 1.4 | 2.4 | 12.1 |
| | HIC | Lithuania | 16.4 | 14.2 | | 7.7 | 8.7 | 17.6 | 1.3 | 1.4 | 3.3 | 16.5 |
| | HIC | Luxembourg | 20.4 | | | 7.2 | 5.6 | 18.9 | 0.7 | 0.5 | 2.1 | 9.5 |
| | HIC | Malta | 5.0 | | | 15.0 | 9.1 | 16.2 | 1.5 | 0.9 | 1.6 | 10.0 |
| | UMIC | Montenegro | 37.0 | | | 22.4 | 23.2 | 36.5 | 1.9 | 2.0 | 3.8 | 8.4 |

| | | | Proportion unrecorded alcohol based on other data sources (%) | | | Proportion unrecorded alcohol based on the statistical model (%) | | | Volume of alcohol *per capita* based on the statistical model (litres of pure alcohol ) | | | |
|---|---|---|---|---|---|---|---|---|---|---|---|---|
| **WHO region** | **Income level** | **Country** | **WHO** | **Experts** | **STEPS** | **% unrecorded** | **95% confidence interval** | | **Unrecorded alcohol** | **95% confidence interval** | | **Total alcohol** |
| | HIC | Netherlands | 5.0 | | | 14.1 | 8.9 | 15.1 | 1.4 | 0.8 | 1.5 | 9.7 |
| | HIC | Norway | 23.0 | | | 15.2 | 12.7 | 25.9 | 1.1 | 0.9 | 2.1 | 7.0 |
| | HIC | Poland | 13.0 | 12.5 | | 13.6 | 10.2 | 16.8 | 1.7 | 1.2 | 2.2 | 12.4 |
| | HIC | Portugal | 13.5 | | | 16.1 | 12.4 | 19.5 | 1.8 | 1.3 | 2.2 | 11.0 |
| | LMIC | Republic of Moldova | 40.0 | 29.3 | 40.1 | 36.6 | 27.6 | 52.9 | 5.5 | 3.6 | 10.7 | 15.1 |
| | UMIC | Romania | 30.0 | 42.5 | | 16.8 | 29.1 | 42.1 | 1.9 | 3.9 | 7.0 | 11.5 |
| | HIC | Russian Federation | 30.0 | 33.4 | | 30.3 | 22.2 | 45.8 | 4.2 | 2.8 | 8.2 | 14.0 |
| | UMIC | Serbia | 23.0 | | | 17.6 | 15.3 | 26.2 | 1.9 | 1.6 | 3.2 | 11.0 |
| | HIC | Slovakia | 14.0 | | | 13.4 | 10.9 | 17.7 | 1.6 | 1.3 | 2.3 | 12.2 |
| | HIC | Slovenia | 10.0 | | | 13.9 | 10.2 | 16.5 | 1.6 | 1.2 | 2.0 | 11.8 |
| | HIC | Spain | 11.0 | | | 16.8 | 12.1 | 19.0 | 1.6 | 1.1 | 1.9 | 9.8 |
| | HIC | Sweden | 19.3 | 24.0 | | 16.5 | 16.9 | 27.3 | 1.4 | 1.4 | 2.7 | 8.5 |
| | HIC | Switzerland | 5.0 | | | 11.2 | 7.1 | 13.2 | 1.2 | 0.7 | 1.4 | 10.6 |
| | LMIC | Tajikistan | 89.0 | | | 70.0 | 69.8 | 85.7 | 0.7 | 0.7 | 1.9 | 1.1 |
| | UMIC | The former Yugoslav Republic of Macedonia | 65.5 | | | 40.9 | 35.1 | 55.1 | 0.7 | 0.5 | 1.2 | 1.6 |
| | UMIC | Turkey | 24.9 | 1.5 | | 36.3 | 10.7 | 20.9 | 0.8 | 0.2 | 0.4 | 2.3 |
| | UMIC | Turkmenistan | 47.0 | | 3.0 | 35.7 | 28.1 | 43.8 | 1.6 | 1.1 | 2.3 | 4.5 |
| | LMIC | Ukraine | 37.0 | 35.7 | | 38.3 | 25.0 | 50.8 | 5.0 | 2.7 | 8.3 | 13.1 |
| | HIC | United Kingdom | 15.0 | | | 11.1 | 9.1 | 17.0 | 1.3 | 1.1 | 2.1 | 11.8 |
| | LMIC | Uzbekistan | 44.0 | | 1.7 | 38.2 | 27.0 | 43.8 | 1.7 | 1.0 | 2.2 | 4.6 |
| SEAR | LMIC | Bangladesh | 95.2 | 85.2 | | 73.0 | 77.1 | 89.2 | 0.0 | 0.0 | 0.1 | 0.0 |
| | LMIC | Bhutan | 52.1 | | 59.1 | 60.9 | 54.5 | 76.0 | 0.4 | 0.3 | 0.7 | 0.6 |

| | | | Proportion unrecorded alcohol based on other data sources (%) | | | Proportion unrecorded alcohol based on the statistical model (%) | | | Volume of alcohol *per capita* based on the statistical model (litres of pure alcohol ) | | | |
|---|---|---|---|---|---|---|---|---|---|---|---|---|
| **WHO region** | **Income level** | **Country** | **WHO** | **Experts** | **STEPS** | **% unrecorded** | **95% confidence interval** | | **Unrecorded alcohol** | **95% confidence interval** | | **Total alcohol** |
| | LIC | Democratic People's Republic of Korea | 13.4 | | | | | | | | | |
| | LMIC | India | 38.0 | 38.0 | | 46.6 | 28.4 | 51.2 | 2.7 | 1.2 | 3.2 | 5.7 |
| | LMIC | Indonesia | 84.7 | | | 63.1 | 63.2 | 82.3 | 0.2 | 0.2 | 0.4 | 0.2 |
| | UMIC | Maldives | 21.6 | | | 58.7 | 26.0 | 59.1 | 2.7 | 0.7 | 2.8 | 4.6 |
| | LMIC | Myanmar | 67.0 | | 11.0 | | | | | | | |
| | LIC | Nepal | 89.0 | 86.6 | | 71.2 | 76.7 | 88.8 | 0.7 | 0.9 | 2.1 | 0.9 |
| | LMIC | Sri Lanka | 38.0 | | 10.0 | 36.5 | 25.7 | 45.9 | 1.5 | 0.9 | 2.1 | 4.0 |
| | UMIC | Thailand | 10.5 | 12.7 | | 21.0 | 9.5 | 22.9 | 1.7 | 0.7 | 1.9 | 8.1 |
| | LMIC | Timor-Leste | 50.0 | | 21.1 | 72.6 | 45.9 | 68.4 | 1.3 | 0.4 | 1.1 | 1.8 |
| WPR | HIC | Australia | 15.0 | | | 7.9 | 6.3 | 14.9 | 0.8 | 0.6 | 1.7 | 10.4 |
| | HIC | Brunei Darussalam | 24.8 | | | 19.3 | 9.8 | 28.1 | 0.2 | 0.1 | 0.4 | 1.2 |
| | LIC | Cambodia | 60.0 | 50.0 | | 47.5 | 41.5 | 64.7 | 1.9 | 1.5 | 3.9 | 4.0 |
| | UMIC | China | 20.0 | 16.8 | | 20.2 | 13.7 | 25.7 | 1.6 | 1.0 | 2.2 | 7.8 |
| | | Cook Islands | 7.3 | | | | | | | | | |
| | UMIC | Fiji | 31.0 | | | 32.0 | 24.4 | 41.9 | 1.1 | 0.7 | 1.6 | 3.3 |
| | HIC | Japan | 3.0 | 3.0 | | 13.9 | 6.5 | 13.0 | 1.2 | 0.5 | 1.1 | 8.8 |
| | LMIC | Kiribati | 80.0 | | | 40.9 | 41.8 | 62.7 | 0.4 | 0.4 | 0.9 | 0.9 |
| | LMIC | Lao People's Democratic Republic | 16.0 | | | 33.0 | 18.4 | 36.0 | 3.0 | 1.4 | 3.4 | 9.1 |
| | UMIC | Malaysia | 67.3 | | | 36.4 | 33.2 | 54.0 | 0.3 | 0.3 | 0.6 | 0.8 |
| | UMIC | Marshall Islands | | | | | | | | | | |
| | LMIC | Micronesia (Federated States of) | 35.0 | | | 36.8 | 28.1 | 47.0 | 0.9 | 0.6 | 1.4 | 2.5 |

| WHO region | Income level | Country | Proportion unrecorded alcohol based on other data sources (%) | | | Proportion unrecorded alcohol based on the statistical model (%) | | | Volume of alcohol *per capita* based on the statistical model (litres of pure alcohol ) | | | |
|---|---|---|---|---|---|---|---|---|---|---|---|---|
| | | | WHO | Experts | STEPS | % unrecorded | 95% confidence interval | | Unrecorded alcohol | 95% confidence interval | | Total alcohol |
| | UMIC | Mongolia | 25.6 | | 1.5 | 22.8 | 15.6 | 28.8 | 1.7 | 1.1 | 2.3 | 7.5 |
| | | Nauru | 69.4 | | | | | | | | | |
| | HIC | New Zealand | 14.0 | | | 12.1 | 9.2 | 18.1 | 1.2 | 0.9 | 1.9 | 9.9 |
| | | Niue | 16.0 | | | | | | | | | |
| | UMIC | Palau | | | | | | | | | | |
| | LMIC | Papua New Guinea | 63.0 | | | 43.8 | 35.5 | 55.3 | 0.7 | 0.5 | 1.1 | 1.6 |
| | LMIC | Philippines | 16.0 | 20.0 | | 31.8 | 17.6 | 31.7 | 2.2 | 1.0 | 2.2 | 6.9 |
| | HIC | Republic of Korea | 21.0 | 5.2 | | 11.3 | 6.6 | 13.5 | 1.2 | 0.7 | 1.5 | 10.6 |
| | LMIC | Samoa | 27.1 | | | | | | | | | |
| | HIC | Singapore | 21.4 | | | 15.7 | 10.0 | 30.7 | 0.3 | 0.2 | 0.8 | 2.2 |
| | LMIC | Solomon Islands | 31.2 | | | | | | | | | |
| | UMIC | Tonga | 30.9 | | | 37.4 | 20.0 | 36.7 | 0.6 | 0.2 | 0.6 | 1.5 |
| | UMIC | Tuvalu | 30.9 | | | | | | | | | |
| | LMIC | Vanuatu | 36.1 | | | | | | | | | |
| | LMIC | Viet Nam | 50.0 | 50.0 | | 31.4 | 35.2 | 53.6 | 2.0 | 2.3 | 5.0 | 6.3 |

WHO=World Health Organization. STEPS=Noncommunicable Disease Risk Factor Survey, part of the STEPwise surveillance (STEPS) by the WHO. AFR=African Region. AMR=Region of the Americas. EMR=Eastern Mediterranean Region. EUR=European Region. SEAR=South East Asian Region. WPR=Western Pacific Region. HIC= High-income country. LIC=Low-income country. LMIC=Lower-middle-income country. UMIC=Upper-middle-income country.

Differences between the modelled estimates and the WHO estimates were highest in Kiribati, Kuwait, Malaysia and Iran, where the modelled estimates were 25% to 40% lower than the WHO estimates. Furthermore, in Israel, Oman, Jordan, Timor-Leste, Maldives, Syrian Arab Republic, and Comoros, the modelled were 20% to 40% higher than the WHO estimates.

**S5 Table. Guidelines for Accurate and Transparent Health Estimates Reporting (GATHER) checklist**

Checklist of information that should be included in new reports of global health estimates

| Item # | Checklist item | Reported on page # |
|---|---|---|
| Objectives and funding | | |
| 1 | Define the indicator(s), populations (including age, sex, and geographic entities), and time period(s) for which estimates were made. | Page 4 |
| 2 | List the funding sources for the work. | Page 12 |
| Data Inputs | | |
| *For all data inputs from multiple sources that are synthesized as part of the study:* | | |
| 3 | Describe how the data were identified and how the data were accessed. | Pages 4-5 |
| 4 | Specify the inclusion and exclusion criteria. Identify all ad-hoc exclusions. | No exclusion |
| 5 | Provide information on all included data sources and their main characteristics. For each data source used, report reference information or contact name/institution, population represented, data collection method, year(s) of data collection, sex and age range, diagnostic criteria or measurement method, and sample size, as relevant. | Pages 4-5, S1 Table, S2 Text |
| 6 | Identify and describe any categories of input data that have potentially important biases (e.g., based on characteristics listed in item 5). | Page 10 |
| *For data inputs that contribute to the analysis but were not synthesized as part of the study:* | | |
| 7 | Describe and give sources for any other data inputs. | Pages 5-6 |
| *For all data inputs:* | | |
| 8 | Provide all data inputs in a file format from which data can be efficiently extracted (e.g., a spreadsheet rather than a PDF), including all relevant meta-data listed in item 5. For any data inputs that cannot be shared because of ethical or legal reasons, such as third-party ownership, provide a contact name or the name of the institution that retains the right to the data. | IHME, WHO |
| Data analysis | | |
| 9 | Provide a conceptual overview of the data analysis method. A diagram may be helpful. | Page 4 |
| 10 | Provide a detailed description of all steps of the analysis, including mathematical formulae. This description should cover, as relevant, data cleaning, data pre-processing, data adjustments and weighting of data sources, and mathematical or statistical model(s). | Pages 4,6 |
| 11 | Describe how candidate models were evaluated and how the final model(s) were selected. | Page 6 |
| 12 | Provide the results of an evaluation of model performance, if done, as well as the results of any relevant sensitivity analysis. | Page 6 |

| | | |
|---|---|---|
| 13 | Describe methods for calculating uncertainty of the estimates. State which sources of uncertainty were, and were not, accounted for in the uncertainty analysis. | Page 6 |
| 14 | State how analytic or statistical source code used to generate estimates can be accessed. | Page 6 |
| Results and Discussion | | |
| 15 | Provide published estimates in a file format from which data can be efficiently extracted. | S4 Table |
| 16 | Report a quantitative measure of the uncertainty of the estimates (e.g. uncertainty intervals). | Table 1, page 8, S4 Table |
| 17 | Interpret results in light of existing evidence. If updating a previous set of estimates, describe the reasons for changes in estimates. | Pages 9-10 |
| 18 | Discuss limitations of the estimates. Include a discussion of any modelling assumptions or data limitations that affect interpretation of the estimates. | Pages 10 |

*This checklist should be used in conjunction with the GATHER statement and Explanation and Elaboration document, found on gather-statement.org*
